# Preoperative Anaemia Increases the Likelihood of 1-Year Mortality After Hip Fracture

**DOI:** 10.1155/aort/5526883

**Published:** 2025-03-29

**Authors:** Helena Ferris, Gavin Sedgwick, Mitchell Marnane, Sean Clarke, Ann Dwyer, Georgia Merron, Tara Coughlan

**Affiliations:** ^1^Department of Public Health, Health Service Executive-South West, Cork, Ireland; ^2^Department of Age-Related Health Care and Orthogeriatrics, Tallaght University Hospital, Dublin, Ireland; ^3^Department of Orthopaedics and Orthogeriatrics, Tallaght University Hospital, Dublin, Ireland; ^4^Discipline of Medical Gerontology, Trinity College Dublin, Dublin, Ireland

**Keywords:** anaemia, haemoglobin, hip fracture, Irish hip fracture standards, long-term outcomes, mortality, survival

## Abstract

**Background:** Preoperative anaemia has been shown to increase the risk of adverse outcomes following hip fracture. To date, the association between haemoglobin (Hb) on admission and longer-term outcomes has not been studied extensively in the Irish hip fracture population. This study aimed to ascertain the mean Hb on admission and investigate the relationship with 1-year mortality.

**Methods:** A retrospective review of all hip fracture cases in older adults discharged from an Irish urban trauma centre over a 4-year period was conducted. Hb on admission was obtained using electronic patient records. Mortality status was obtained via the Inpatient Management System and correlated with the Irish Death Events Register. Logistic regression was performed on a range of routinely collected patient and care pathway variables.

**Results:** A total of 833 patients were included. Mean Hb on admission was 12.1 g/dL (SD 1.7), with 11.4% (95/833) of patients having a Hb ≤ 10 g/dL. Within 1 year of fracture 20.5% (171/833) of patients had died. On multivariate analysis, those with a Hb ≤ 10 g/dL on admission were 76% more likely to die within one year (OR 1.76, *p* < 0.02, 95% CI 1.07–2.90) compared to those with a Hb ≥ 10.1 g/dL. Patients admitted from a nursing home were also more likely to die within 1 year (OR 2.09, *p* < 0.001, 95% CI 1.26–3.45), compared to those admitted from home. Early postoperative mobilisation (OR 0.32, *p* < 0.001, 95% CI 0.22–0.48) and female gender (OR 0.49, *p* < 0.001, 95% CI 0.34–0.71) reduced the likelihood of 1-year mortality (AUC 0.71).

**Conclusion:** Anaemia is common in hip fracture patients and may be viewed as a surrogate marker of frailty. In this cohort, Hb ≤ 10 g/dL on admission was a statistically significant predictor of 1-year mortality. Recognising and managing anaemic patients preoperatively may provide an opportunity to improve longer-term outcomes in hip fracture patients.

## 1. Introduction

Anaemia in older surgical patients is associated with increased mortality, prolonged length of hospital stay and poorer functional outcomes [1]. According to the World Health Organization (WHO), anaemia is defined as haemoglobin (Hb) < 12.0 g/dL in women and < 13.0 g/dL in men [2]. Preoperative anaemia is common in hip fracture patients with a prevalence of approximately 42% (ranging from 17% to 61%) [3–7]. The aetiology of this can be multifactorial i.e., trauma-induced blood loss, iron deficiency, anaemia secondary to chronic comorbidities, bone marrow dysfunction, etc [8]. Most recently, the International Consensus Conference on Anaemia Management in Surgical Patients (ICCAMS) recommended that all patients, except those undergoing a minor procedure, should be screened for anaemia prior to surgery and cause appropriate treatment initiated [9]. Irrespective of the underlying pathology, identifying and managing low Hb preoperatively could provide an opportunity to improve patient outcomes.

Anaemia on admission is associated with inferior outcomes after hip fracture. A systematic review of 54 studies by Potter et al. found a consistent association between anaemia on admission and increased postoperative mortality (RR 1.64, 95% CI 1.47–1.82, *p* < 0.001) [10]. Similarly, a study of 34,805 patients in the American College of Surgeons National Surgical Quality Improvement Programme (ACS-NSQIP), demonstrated that anaemia at presentation was independently associated with 30-day mortality (OR 1.3, 95% CI 1.1–1.5) and readmission (OR 1.2, 95% CI 1.1–1.3) [11]. Moreover, a 20-year observational study of a European cohort of 3595 patients showed that basal Hb was a useful and cost-effective parameter for predicting early mortality in hip fracture patients. Kovar et al. showed that anaemia at admission was associated with an increase in 3-month mortality when adjusted for age and sex [mild anaemia aOR 1.5 (95% CI 1.1–1.9), moderate anaemia aOR 2.6 (95% CI 2.0–3.4) and severe anaemia aOR 3.6 (95% CI 1.8–6.9)] [12].

In relation to morbidity, Bailey et al. demonstrated that anaemic patients were more likely to experience post-operative complications (OR 1.42, 95% CI 0.99–2.03, *p* < 0.05), require a transfusion (OR 4.09, 95% CI 2.64–6.35, *p* < 0.001) and have a longer length of stay (OR 1.19, 95% CI 1.13–1.26, *p* < 0.001) [13]. More specifically, preoperative Hb < 10 g/dL has been shown to predict Major Adverse Cardiac Events (OR 1.79, 95% CI 1.07–2.98, *p* < 0.02) and in-hospital mortality (OR 2.81, 95% CI 1.2–6.5, *p* < 0.01) in hip fracture patients over 80 years [14]. Anaemia has also been shown to impede functional mobility after hip fracture surgery and is an independent risk factor for patients not being able to walk on the third postoperative day (OR 0.41, 95% CI 0.14–0.73, *p*=0.002) [15].

## 2. Aim

The primary aim of this study was to ascertain the mean Hb on admission in an Irish hip fracture cohort and investigate the relationship between preoperative Hb and 1-year mortality. A secondary objective was to investigate the association between routinely collected variables and longer-term mortality.

### 2.1. Methods

**Figure 1:**
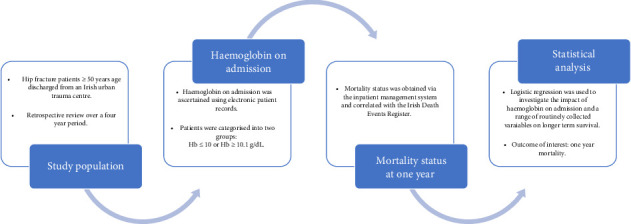
Overview of methodology.

The authors conducted a review of older adults with hip fracture in an Irish urban trauma centre over a 4-year period (Figure 1). Data were collected retrospectively for all hip fracture patients over 50 years of age discharged from Tallaght University Hospital (TUH) from 01/01/2018 to 31/12/2021. Details of the episode of care were entered into the hospital's hip fracture repository using information from the Hospital Inpatient Enquiry (HIPE) system. This system records hip fracture cases with a HIPE Code S72.0–S72.2 or with a specified fracture type i.e., intracapsular displaced, intracapsular undisplaced, intertrochanteric, subtrochanteric or periprosthetic. Pathological fractures are excluded. TUH is an academic teaching hospital and one of 16 trauma units nationally that submit data from its hospital repository to the Irish Hip Fracture Database using a standardised data collection proforma [16]. This captures evidence-based variables of interest ranging from patient demographics to preoperative functional status and care process measures, in keeping with the minimum common dataset recommended by the Fragility Fracture Network [17].

Electronic hospital records were used to ascertain Hb on admission, which was measured for all patients as part of the preoperative clinical assessment and laboratory investigations. The cohort was categorised into two groups Hb ≤ 10.0 and Hb ≥ 10.1 g/dL in keeping with the literature and validated scoring systems that incorporate Hb as a marker of physiological reserve [18, 19]. Mortality status was obtained electronically via the Inpatient Management System and correlated with the Irish Death Events Register.

### 2.2. Analysis

Statistical analysis was conducted using Stata® (version 18). Descriptive statistics were presented as mean and standard deviation or frequency and percentage, as appropriate. A range of routinely collected variables capturing patient factors and the care pathway were analysed initially using univariate logistic regression. A multivariate logistic regression model was constructed using the statistically significant variables from univariate analysis to assess the likelihood of dying within 1 year of fracture. Odds Ratios (OR) and 95% Confidence Intervals (CI) were used to describe the strength of the association. A value of *p* < 0.05 indicated statistical significance throughout. The predictive accuracy of the regression model was determined by using Area Under the Curve (AUC) statistics.

## 3. Results

### 3.1. Descriptive Statistics

**Figure 2:**
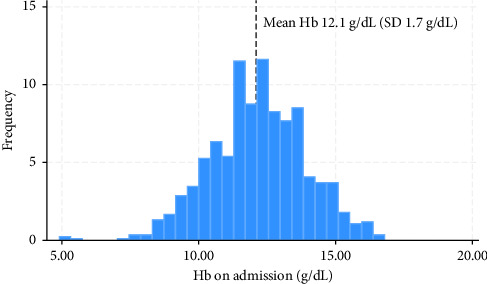
Distribution of Hb on admission in hip fracture patients 2018–2021.

The retrospective review consisted of 833 hip fracture patients. Data pertaining to Hb on admission and 1-year mortality was obtained for 100% of cases. Mean Hb on admission for the cohort was 12.1 g/dL (SD 1.7) (Figure 2), with 11.4% (95/833) of patients having a Hb ≤ 10 g/dL on admission.

**Figure 3:**
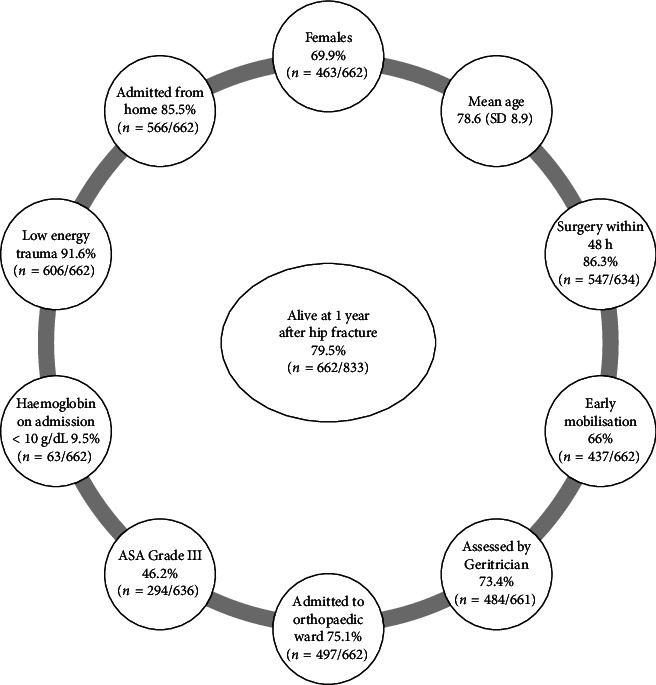
Characteristics of patients alive at 1 year after hip fracture.

Mean age was 78.6 years (SD 8.9) with females accounting for 67% (556/833) of the cohort. Home was the most frequent source of admission with almost one-third of patients having previously sustained a fragility fracture [28.7% (239/833)]. The most common fracture type was intertrochanteric [41.7% (347/831)], followed by intercapsular displaced [29.2% (243/831)]. Cemented bipolar hemi arthroplasty was the most common type of surgical intervention [35.2% (294/833)], followed by internal fixation with a short IM nail [24.1% (201/833)]. Surgery was typically carried out using spinal anaesthesia alone [46.5% (366/787)], followed by general anaesthesia with a nerve block [17.9% (141/787)]. Almost half of patients had severe systemic disease corresponding to American Society of Anaesthesiologists (ASA) Grade III [48.2% (380/787)]. The majority of patients [84% (662/787)] were operated on within 48 h of admission, with 59.6% (497/833) of patients mobilised early in the postoperative period. Mean Length of Stay (LOS) was 22.3 days (SD 32.5). Mortality at 1 year was 20.5% (171/833). Of those who died within one year, 18.7% (32/171) had Hb ≤ 10 g/dL compared to 9.5% (963/662) who were alive at one year.  Figure 3 outlines the characteristics of those alive at 1 year following hip fracture.

### 3.2. Analytical Statistics

On univariate analysis, those with a Hb ≤ 10 g/dL on admission were more than twice as likely to die within one year of hip fracture (OR 2.18, *p* < 0.001, 95% CI 1.37–3.48) compared to those with a Hb ≥ 10.1 g/dL (Table 1). Female gender (OR 0.51, *p* < 0.001, 95% CI 0.36–0.72) and early postoperative mobilisation (OR 0.29, *p* < 0.001, 95% CI 0.20–0.43) were protective, whereas being admitted from a nursing home increased the likelihood of dying within 1 year of fracture (OR 2.58, *p* < 0.001, 95% CI 1.61–4.13).

The statistically significant variables from univariate analysis were included in a multivariate model. On multivariate analysis, those with a Hb ≤ 10 g/dL on admission were 76% more likely to die within one year of fracture (OR 1.76, *p* < 0.02, 95% CI 1.07–2.90) compared to those with a Hb ≥ 10.1 g/dL (Table 2). Patients who were admitted from a nursing home were also more likely to die within 1 year (OR 2.09, *p* < 0.001, 95% CI 1.26–3.45), compared to those admitted from home. Conversely, female gender (OR 0.49, *p* < 0.001, 95% CI 0.34–0.71) and early postoperative mobilisation (OR 0.32, *p* < 0.001, 95% CI 0.22–0.48) reduced the likelihood of 1-year mortality.

**Figure 4:**
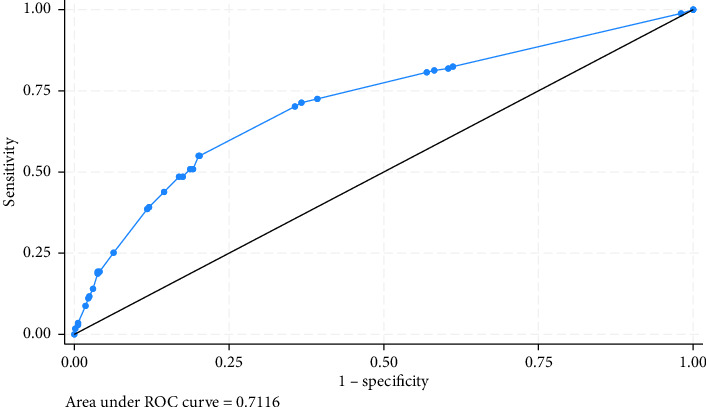
Area under curve analysis.

The model had a good discriminating ability with an AUC of 0.71 (Figure 4).

## 4. Discussion

**Figure 5:**
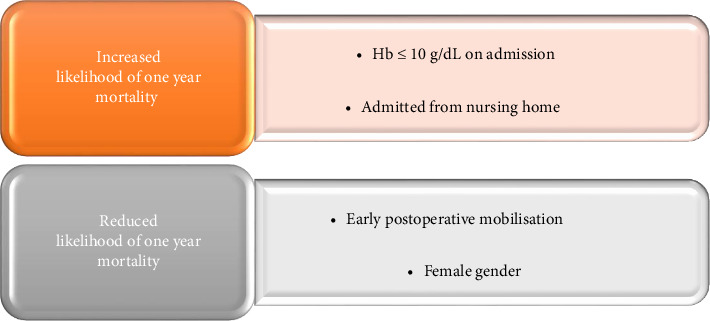
Main study findings.

Anaemia is common in older adults and is associated with reduced functional capacity, frailty and an increased risk of fracture [20, 21]. This paper investigated the association between Hb on admission and 1-year mortality in an Irish hip fracture cohort. The results of the multivariate logistic regression analysis showed that Hb ≤ 10 g/dL (OR 1.76, *p* < 0.02, 95% CI 1.07–2.90) and admission from a nursing home (OR 2.09, *p* < 0.001, 95% CI 1.26–3.45) increased the likelihood of dying within one year of fracture, whereas female gender (OR 0.49, *p* < 0.001, 95% CI 0.34–0.71) and early mobilisation (OR 0.32, *p* < 0.001, 95% CI 0.22–0.48) reduced the risk of death (AUC 0.71) (Figure 5). Of the 4 statistically significant predictors of 1-year mortality, only two variables were modifiable factors in the care pathway, namely preoperative Hb and early postoperative mobilisation.

Hb on admission is a component of several scoring systems designed to predict recovery following hip fracture. For example, the Nottingham Hip Fracture Score (NHFS) incorporates Hb ≤ 10 g/dL, the Orthopaedic Physiological and Operative Severity Score for the enUmeration of Mortality and Morbidity (O-POSSUM) and Amelo Hip Fracture Score (AHFS) also include Hb on admission as a variable in the risk prediction model [22–24]. Identifying preoperative anaemia could provide a readily available and cost-effective target for intervention, given that most patients will have a documented Hb as part of their preoperative laboratory investigations.

The Association of Anaesthetists' guidelines for the management of hip fracture recommend that perioperative Hb in frail patients should be kept above 9 or 10 g/dL in patients with a history of ischaemic heart disease [25]. Treatment strategies may include the administration of iron, with or without erythropoietin, minimising intraoperative blood loss or blood transfusion [26–28]. However, it is important to balance the risks of anaemia against the possible complications associated with treatment. Red blood cell transfusion in particular requires careful consideration, as there are pros and cons to both restrictive and liberal transfusion strategies [29]. Furthermore, the optimal timing of the correction of anaemia has long been the subject of debate i.e., preoperative, intraoperative or postoperative. Ideally, any correctable comorbidities such as anaemia should be identified and treated as early as possible to enable timely surgical intervention [30, 31]. However, deciding on the best blood management strategy can be challenging, particularly in the context of hip fracture being a major trauma that requires urgent care. In consideration of the complex needs of older hip fracture patients and the nuanced nature of decisions pertaining to blood management, the Fragility Fracture Network Guidelines and the Association of Anaesthetists guidelines recommend that the management of perioperative anaemia should be carried out in accordance with agreed hospital protocols.

Anaemia can also hinder rehabilitation in the early postoperative phase [32]. This is important given the beneficial effect of early ambulation on both short and longer-term mortality in hip fracture patients [33]. The analysis presented in this paper demonstrated that patients who were stood out of bed at minimum by a Physiotherapist on the first postoperative day were 68% less likely to die within 1 year of fracture (OR 0.32, *p* < 0.001, 95% CI 0.22–0.48). In this cohort, 59.6% (497/833) of patients were mobilised early in the postoperative period, which is lower than the current national average of 86%. However, early mobilisation was added as a formal standard of care to the Irish Hip Fracture Standards in 2020, which is halfway through the time period incorporated in this cohort. TUH identified this as an area for improvement and in 2022, 82.6% (180/218) of patients achieved this national standard of care.

Prefracture residence was also shown to impact 1-year survival. Patients who were admitted from a nursing home were more likely to die within 1 year (OR 2.09, *p* < 0.001, 95% CI 1.26–3.45), compared to those admitted from home. According to The Irish Longitudinal Study on Ageing (TILDA), the prevalence of frailty in community-dwelling adults over 75 years is 25% [34]. However, this figure rises to 68% in nursing home residents [35]. These are a particularly vulnerable subset of patients who are at an increased risk of falls, cogitative impairment, depression, medication side effects, etc. In recent years, there has been an increased focus on supporting care homes in implementing targeted interventions such as strength training and falls prevention programs. A recent systematic review by Pinheiro et al. showed that multicomponent or resistance training improved physical performance in institutionalised older adults (*p* < 0.05) [36]. Furthermore, a systematic review by Cordes et al. showed that Chair Based Interventions may improve not only physical function but also cognitive function and general well-being in nursing home residents [37]. Once admitted to the acute hospital, nursing home patients may benefit from being prioritised for preoperative assessment by an orthogeriatrician. However, there is a window of opportunity to move from a reactive to a proactive approach in this high-risk group of patients. Strengthening links between community, primary and secondary care could allow patients to be identified in the prefrail state to work towards reducing their vulnerability to adverse outcomes.

In this cohort, the most common fracture type was intertrochanteric (41.7%), with cemented bipolar hemi arthroplasty being the most common type of surgical intervention (35.2%). There is variation both nationally and internationally in terms of surgical intervention for each fracture subtype. A recent overview of trends in Irish hip fracture surgery by Irwin et al. highlighted a decline in the use of DHS for intertrochanteric fractures with an increased use of IM nails and an overall low rate of total hip replacement for intracapsular fractures [38]. The evidence base is evolving in relation to the optimal surgical intervention for various patient groups with particular emphasis on outcomes such as pain, mobility, revision rates and mortality.

The mean LOS of 22 days was longer than the national average of 19 days [39]. However, it is important to note that case mix varies between the 16 trauma units nationally in terms of age, degree of frailty, admission source etc. Furthermore, the analysis presented in this paper provides valuable insights into longer-term survival following hip fracture. One-year mortality in this cohort was 20.5% and is comparable to other Irish trauma units which recently reported 1-year mortality of 20.8% [40, 41]. Given the increased focus on living well for longer, it is imperative that we follow patients beyond the in-hospital journey to better understand how we can support them in living full independent lives after the acute phase of treatment.

At its most basic level, this paper highlights that anaemia at time of admission is a good indicator of poor general health status and susceptibility to negative outcomes [42, 43]. Not only does it impact survival but it has also been shown to impair physical function and Health Related Quality of Life (HRQOL) long after the patient is discharged from hospital [44, 45]. Early detection and management of anaemia could enhance recovery, aid rehabilitation and improve outcomes for patients.

## 5. Limitations

This paper explored the relationship between preoperative Hb and longer-term outcomes following hip fracture. To our knowledge, this is the first time the association between preoperative anaemia and 1-year mortality has been demonstrated in an Irish hip fracture cohort. However, there are limitations to the work presented. Firstly, this study is retrospective and utilised observational data, therefore the authors cannot draw any conclusion in relation to causality. In addition, the paper focussed on Hb on admission and did not have information pertaining to postoperative Hb or the impact of any treatment strategy employed. Moving forward, outcomes other than survival need to be considered. Functional outcomes such as mobility are important to consider as they are a key component of quality of life.

## 6. Conclusion

Anaemia is a useful composite indicator of a patient's general health status, which can signal frailty and an increased vulnerability to negative outcomes. It can hinder postoperative rehabilitation and increase the risk of death. The analysis presented in this paper showed that Hb ≤ 10 g/dL on admission is a statistically significant predictor of 1-year mortality. Of the variables examined, early postoperative mobilisation was the only other modifiable factor identified. Identifying and managing low Hb preoperative, in consultation with the multidisciplinary team involved in the care of older adults, may be a meaningful target for intervention.

## Figures and Tables

**Table 1:** Univariate logistic regression.

Variable	Odds ratio	*p* value	95% CI
Sex			
Male	1 (base)		
Female	0.51	< 0.001	0.36–0.72
Age			
50–59	1 (base)		
60–69	0.07	0.07	0.00–1.27
70–79	0.20	0.26	0.01–3.37
80–89	0.33	0.44	0.02–5.41
90–99	0.56	0.68	0.03–9.28
100–109	1.99	0.71	0.05–78.24
Hb on admission ≤ 10 g/dL			
No	1 (base)		
Yes	2.18	< 0.001	1.37–3.48
Fracture type			
Intertrochanteric	1 (base)		
Intracapsular-displaced	0.67	0.06	0.44–1.02
Intracapsular-undisplaced	0.82	0.41	0.52–1.30
Not documented/other	0.91	0.88	0.24–3.34
Periprosthetic	0.50	0.27	0.14–1.72
Subtrochanteric	1.26	0.53	0.60–2.64
Admission source			
Home	1 (base)		
Transfer from another hospital	0.98	0.97	0.46–2.08
Transfer from nursing home	2.58	< 0.001	1.61–4.13
Medical card⁣^∗^			
No	1 (base)		
Yes	1.10	0.58	0.76–1.61
Admitted to orthopaedic ward			
No	1 (base)		
Yes	0.80	0.25	0.55–1.16
Surgical delay			
Awaiting inpatient or high-dependency bed	1 (base)		
Awaiting medical review/investigation	3.33	0.28	0.36–30.71
Awaiting orthopaedic diagnosis/investigation	0.41	0.56	0.02–8.05
Awaiting space on theatre list	1.66	0.74	0.07–37.72
Issues due to anticoagulation	1.25	0.88	0.05–26.8
No delay-surgery < 48 h	1.05	0.96	0.12–9.08
Not documented/other	1.66	0.67	0.17–15.85
Assessed by geriatrician			
No	1 (base)		
Yes	0.92	0.70	0.63–1.35
Mobilised early			
No	1 (base)		
Yes	0.29	< 0.001	0.20–0.43
Unknown	1.61	0.13	0.85–3.02
ASA grade	1.07	0.11	0.98–1.17
Length of stay	1.01	< 0.001	1.00–1.01

⁣^∗^A medical card allows patients under a specific income threshold to access primary care, community health services, dental services, prescription medicines and hospital care free of charge.

**Table 2:** Multivariate logistic regression.

Variable	Odds ratio	*p* value	95% CI
Hb ≤ 10 g/dL			
No	1 (base)		
Yes	1.76	0.02	1.07–2.90
Admission source			
Home	1 (base)		
Transfer from another hospital	0.95	0.91	0.43–2.10
Transfer from nursing home	2.09	< 0.001	1.26–3.45
Sex			
Male	1 (base)		
Female	0.49	< 0.001	0.34–0.71
Mobilised early			
Yes	0.32	< 0.001	0.22–0.48
No	1.72	0.10	0.89–3.31

## Data Availability

The data that support the findings of this study are available on request from the corresponding author. The data are not publicly available due to privacy or ethical restrictions.
